# Full Mouth Rehabilitation for a Patient With Generalized Attrition: The Hobo Technique in Action

**DOI:** 10.7759/cureus.51933

**Published:** 2024-01-09

**Authors:** Lisa Debbarma, Vineet Sharma

**Affiliations:** 1 Prosthodontics, Rajasthan University of Health Sciences College of Dental Sciences, Jaipur, IND

**Keywords:** patient-centered, vertical dimension, attrition, pfm crowns, hobo twin-stage

## Abstract

This case report addresses the critical issue of severe tooth wear and its impact on the vertical dimension of occlusion in a 75-year-old patient. The patient presented with worn-down teeth, chewing difficulties, and aesthetic concerns, prompting a comprehensive treatment plan utilizing the Hobo twin-stage technique. The approach involved careful diagnosis, root canal therapy, and the creation of provisional restorations, monitored through an occlusal splint. The prosthetic phase incorporated strategically designed porcelain-fused-to-metal crowns, aiming for optimal tooth alignment and balanced movement. The successful outcome not only restored function and aesthetics but also enhanced the patient's confidence. This case underscores the effectiveness of a systematic approach in managing severe tooth wear, emphasizing the importance of regular follow-ups and good oral hygiene for long-term success.

## Introduction

Throughout the course of an individual's life, the occlusal surfaces of their teeth undergo a natural process of gradual erosion or wear [1]. This wear on tooth structure represents an irreversible condition, and when it reaches an excessive level, it can give rise to a host of dental issues. Excessive tooth material loss not only poses risks of pulpal pathology but also contributes to occlusal disharmony, diminished function, aesthetic deformity, and, eventually, psychological discomfort [2]. The factors contributing to excessive wear can be diverse, ranging from congenital or developmental conditions like amelogenesis imperfecta and dentinogenesis imperfecta to acquired issues such as attrition, abrasion, and erosion [3]. Managing patients with significant wear often necessitates extensive restorative treatments.

In the year 1984, Turner and Missirlian introduced a comprehensive three-category classification system for occlusal wear. This classification discerns cases with excessive wear and accompanying loss of occlusal vertical dimension (Category 1), those with excessive wear but without a loss of vertical dimension yet with available space (Category 2), and cases characterized by excessive wear without a loss of vertical dimension but within limited space (Category 3). To address the complexities associated with these categories, various full-mouth rehabilitation philosophies have been established, including the Hobo twin table, Hobo twin stage, and Pankey Mann Schuyler approaches [4]. Each of these philosophies is meticulously designed to cater to specific wear categories. In this context, Porcelain-Fused-to-Metal (PFM) crowns emerge as a recognized and effective modality for replacing missing tooth structures, offering a dual benefit of functional restoration and aesthetic enhancement [5].

In the realm of this clinical report, we delve into the application of the Hobo twin-stage technique. This approach is strategically employed to address a patient grappling with a reduced vertical dimension of occlusion (VDO) and generalized attrition. The overarching goal is to elevate oral health and enhance the quality of life by restoring not only aesthetics but also functionality and occlusal harmony [6]. The Hobo twin-stage technique, with its systematic approach, provides a tailored solution to the intricate challenges posed by reduced VDO and generalized attrition. By implementing this technique, comprehensive rehabilitation seeks to rejuvenate the patient's oral health and overall well-being.

## Case presentation

**Figure 1:**
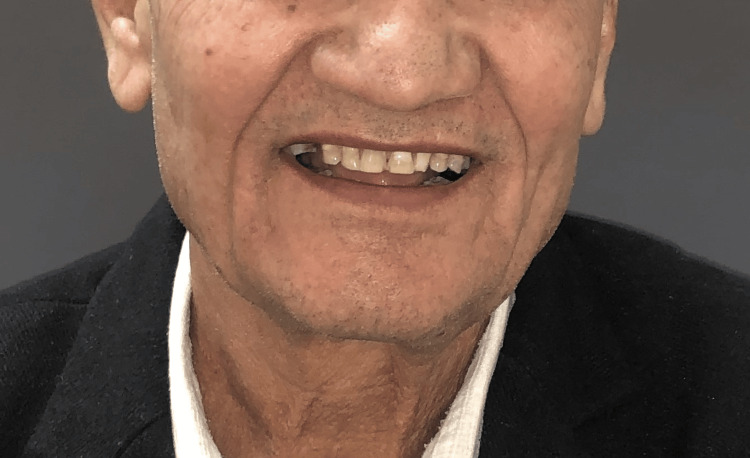
Preoperative extraoral portrait view

A male patient, age 75, arrived at the prosthodontics department with chief complaints of worn-down teeth, difficulty chewing, and aesthetic concerns (Figure 1). His medical history revealed no relevant issues. The patient demonstrated cooperation and expressed a willingness to undergo treatment.

**Figure 2:**
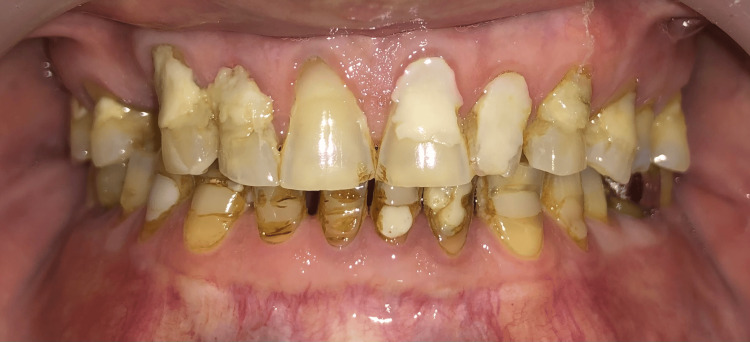
Preoperative photograph showing generalized attrition and restored cervical attrition

**Figure 3:**
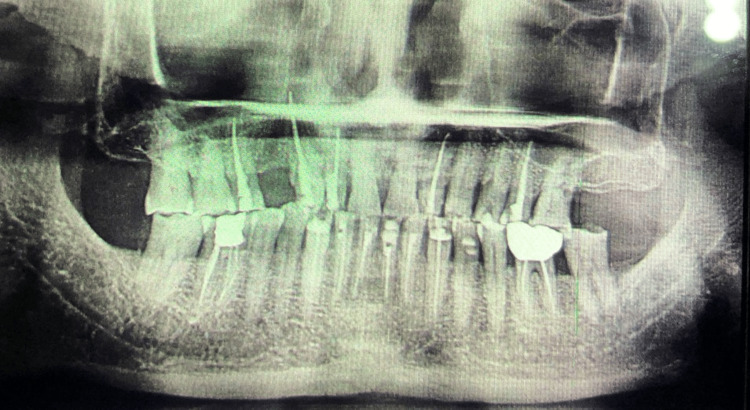
Panoramic radiograph showing the requirement of root canal treatment

The patient had a reduced VDO and generalized attrition affecting all of the teeth, according to a clinical examination, along with generalized cervical abrasion, which was restored by composite resin (Figure 2). The patient had missing teeth with respect to (w.r.t.) 14, 27, and a single unit metal crown w.r.t. 36 (root canal treated) with an exposed margin. A radiograph examination revealed the requirement of root canal treatment for 12, 13, 15, 22, 24, 25, 31, 32, 33, 34, 35, 41, 42, 43, and 46 (Figure 3). The patient's temporomandibular joint was evaluated and reported to be normal, with no evidence of pain or discomfort. Following clinical assessment, it was established that restoring the VDO by increasing it by 2 mm was viable. The patient was told of the diagnosis and given a thorough description of the treatment plan.

**Figure 4:**
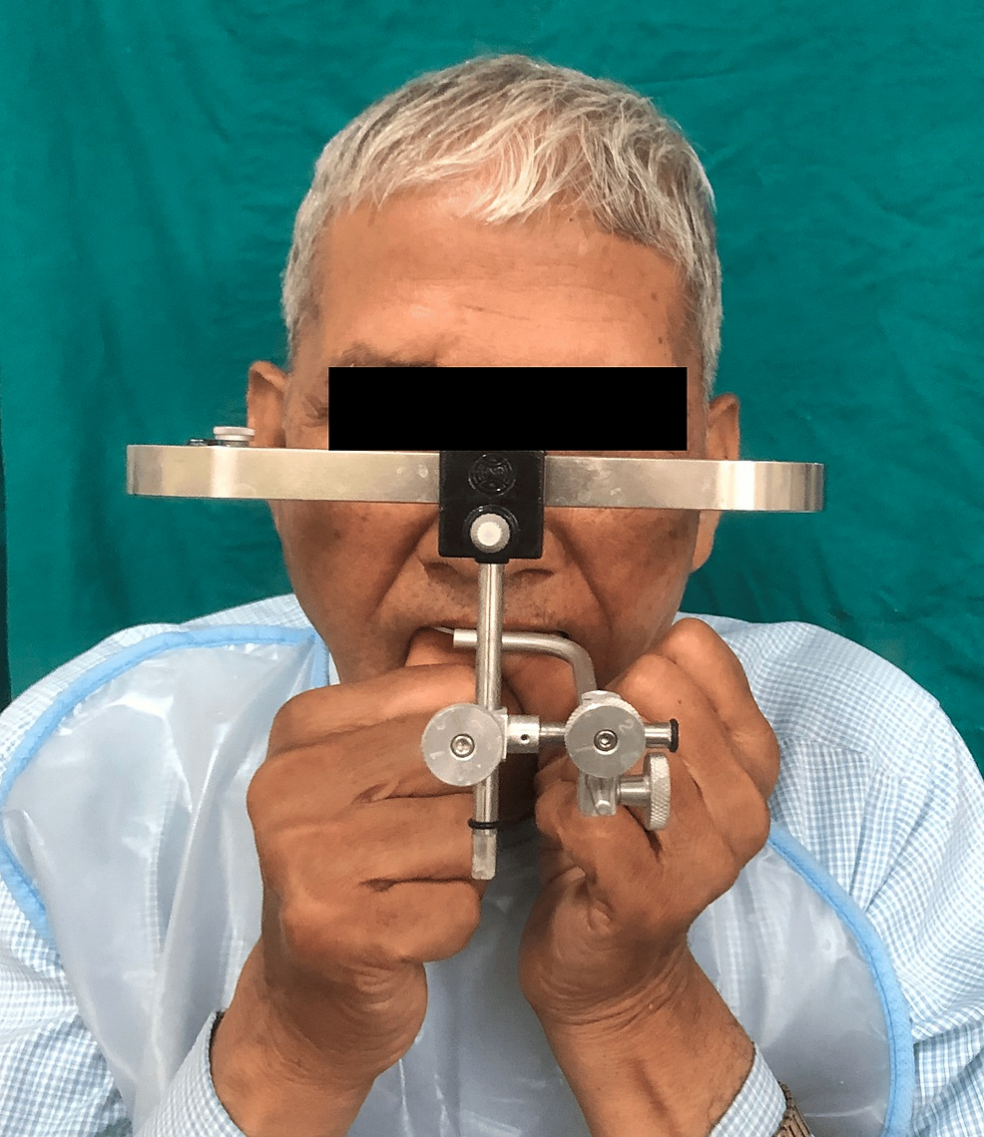
Facebow record

The treatment began after informed consent was obtained. Primary impression with irreversible hydrocolloid impression material (WaldentFlexiPrint Alginate, WaldentAlChem, New Delhi, India) were made to create diagnostic casts. The Hanau spring bow recorded the orientation jaw relation, and Dawson's technique captured the centric relation (CR) (Figure 4). These records were then transferred to a Hanau Wide Vue semi-adjustable articulator. An occlusal splint was then fabricated at an increased VDO. This splint ensured consistent tooth contact in CR and disocclusion of posterior teeth during eccentric movements.

**Figure 5:**
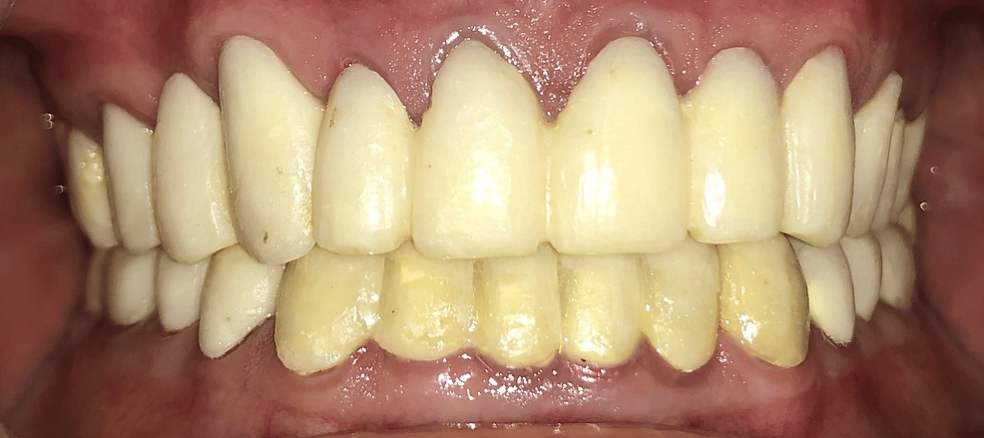
Provisional crowns in situ

**Figure 6:**
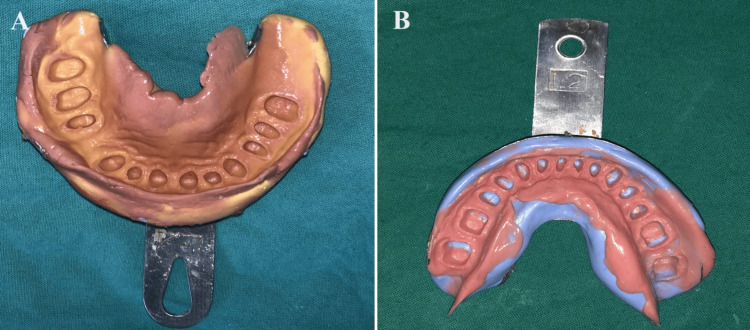
Master impression: (A) Maxillary Arch and (B) mandibular arch

The patient was directed to the endodontic department to undergo root canal therapy. The adaptation to the recently increased VDO was monitored using an occlusal splint for one month, during which there were no indications of temporomandibular joint discomfort or muscle tenderness. The diagnostic wax-up was finalized, maintaining the increased VDO on the mounted diagnostic cast. Subsequently, a putty index was made. Following the standard PFM restoration protocol, minimal occlusal reduction was done during teeth preparation. Provisional crowns were then fabricated using the putty index derived from the diagnostic wax-up. Before cementation with provisional luting cement (Temlute, Eugenol free Temporary luting cement, Prime, Maharashtra, India) the provisional fixed restorations were scrutinized for aesthetics and phonetics (Figure 5). The adaptation of these provisional restorations was assessed over four weeks. Following this period, an impression of the entire arch was obtained using elastomeric impression material (GC Flexceed; GC India, Telangana, India) (Figure 6A, 6B).

**Figure 7:**
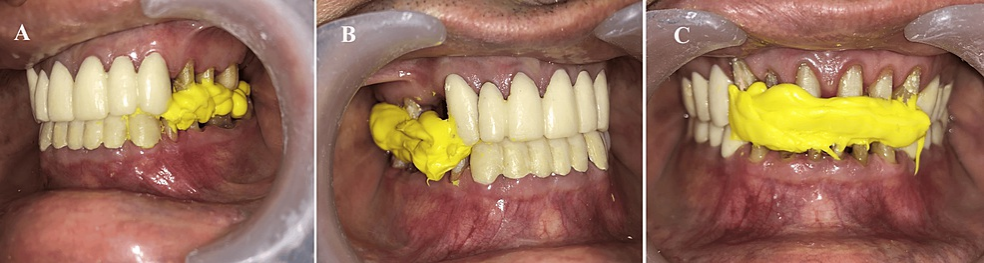
Vertical and centric record transfer: (A) Left side, (B) right side, and (C) front side

**Figure 8:**
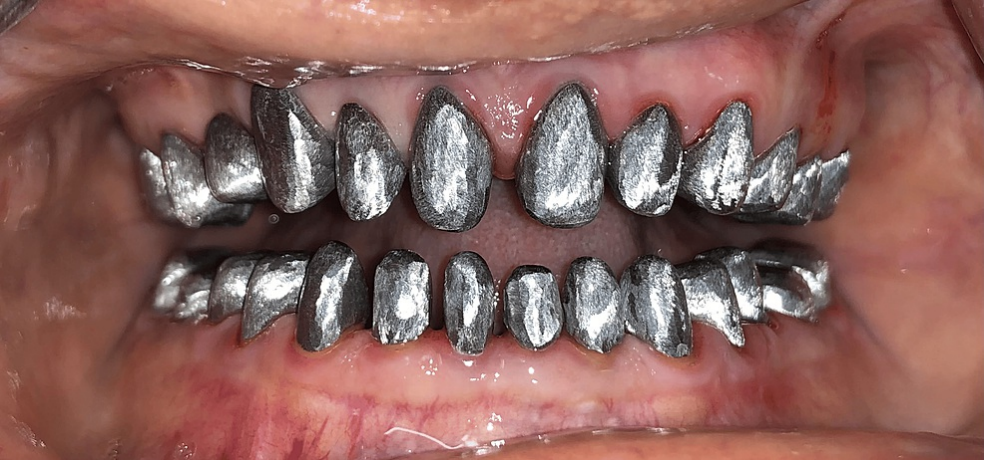
Metal copings try in

Following the facebow record, the working casts were mounted onto a semi-adjustable articulator, utilizing the facebow record for precise alignment. To accurately transfer the VDO and CR, three segmental interocclusal records were obtained using bite registration material. This involved removing the provisional crown from the left segment and placing the bite registration material (JET BITE, Coltene, Maharashtra, India) in that specific area while retaining the provisional crowns in the right and anterior segments. The right and anterior segments underwent a similar process (Figures 7A-7C). Subsequent to making wax patterns, metal copings were fabricated and evaluated in the patient's mouth to ensure a proper fit, which was subsequently confirmed by the working cast (Figure 8).

**Figure 9:**
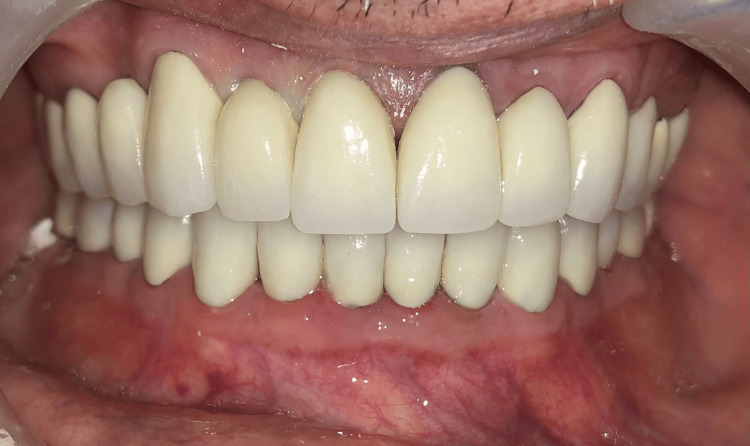
Final prostheses in situ

The porcelain build-up was conducted in two stages. In the initial phase, the anterior section of the cast was removed. Adjustments were then made to the condylar and incisal guidance on the articulator to align with Hobo's condition 1, ensuring standardized effective cusp angles. The occlusal morphology of the posterior teeth was designed to establish contact between the maxillary and mandibular cusps during eccentric movements, aiming for balanced articulation with standardized cusp angles for each individual cusp. In the subsequent phase, the anterior section was again placed to the cast, and modifications were made to achieve Hobo's condition 2, with a focus on posterior disocclusion. The porcelain build-up was carried out to facilitate contact between maxillary and mandibular incisors during protrusive movements and between maxillary and mandibular canines on the working side during lateral movements. This helped establish anterior guidance and the necessary disocclusion. Finally, the PFM crowns were cemented, and subsequent occlusal adjustments were performed to ensure adequate contact and functionality (Figure 9).

**Figure 10:**
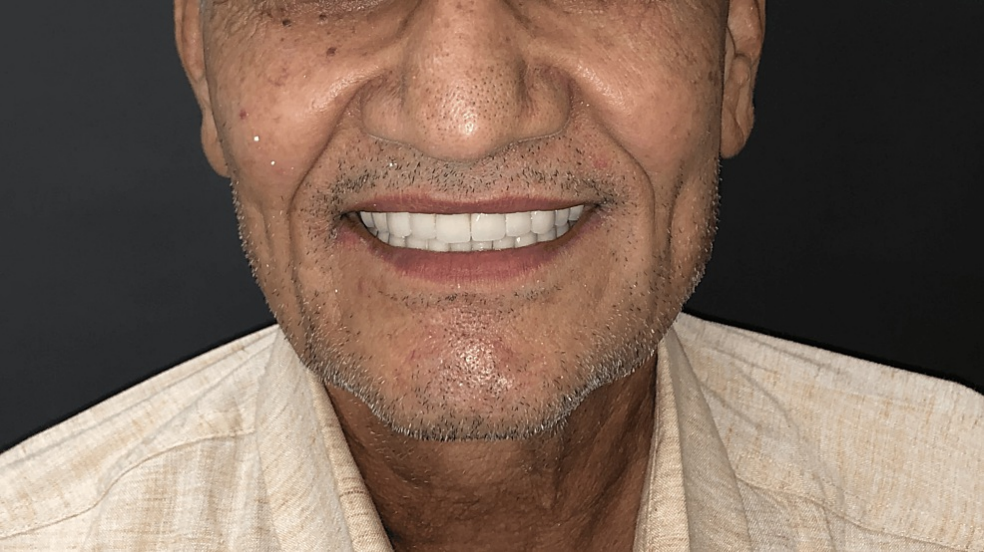
Smile of satisfaction

Periodic check-ups and instructions on maintaining good oral health were provided to assess healing and ensure the long-term success of the treatment. The patient expressed satisfaction with the outcome, noting fulfilment in both the aesthetics and function of the teeth, which, in turn, restored his confidence and dignity (Figure 10).

## Discussion

The prevalence of generalized attrition among older adults brings forth an array of functional, aesthetic, and occlusal health challenges that significantly impact their overall well-being. Attrition is typically mitigated by the compensatory elongation of the alveolar process, a consequence of gradual alveolar bone remodeling [7]. This compensatory mechanism plays a crucial role in maintaining the VDO, preserving the harmonious alignment of dental arches. However, instances arise where wear outpaces continuous eruption, resulting in an excessive loss of VD and contributing to issues such as reduced aesthetics, discomfort during chewing, and an overall decline in quality of life [8].

To fortify preventive and restorative care, it is crucial to identify the cause of wear before intervention [9]. Various assessment methods, including phonetics, aesthetics, and interocclusal distance evaluation, prove invaluable in confirming VDO loss [10]. In the specific case under consideration, a meticulous examination uncovered a significant 4mm loss in VDO. The necessary bite rise was accurately calculated using sophisticated methods like the “closest speaking space” and a thorough assessment of the “freeway space.”

In the realm of dental interventions, PFM crowns emerge as an effective and aesthetically pleasing solution to restore worn-out tooth structure. This approach not only addresses functional aspects but also enhances the overall aesthetic appeal of the patient's teeth. The decision to opt for PFM crowns is grounded in their inherent qualities, including excellent marginal adaptability and surface finish, significantly contributing to the rehabilitation's durability.

The chosen rehabilitation method for the patient, who presented with considerable generalized attrition resulting in a diminished VDO, involved the implementation of the Hobo twin-stage technique [11-13]. This technique places a strong emphasis on creating anterior guidance to achieve predetermined and harmonious posterior disocclusion along the condylar path. Noteworthy benefits of this technique include its simplicity, ease of learning, and the predictability of practical and aesthetic outcomes [14,15]. Furthermore, it offers a high degree of flexibility and customization, tailoring the treatment to meet the unique needs of individual patients, all while being less time-consuming.

Following the comprehensive rehabilitation, the patient expressed contentment with both the aesthetic outcomes and the improvement in oral health. However, it is essential to underscore the significance of regular follow-up visits and diligent dental hygiene maintenance, as they are imperative for ensuring the long-term success of the treatment. As we reflect on the intricate journey of dental care, it is imperative to acknowledge the dynamic nature of oral health management, particularly within the context of an aging population. Each case presents a unique tapestry of challenges, demanding a judicious blend of diagnostic precision, evidence-based interventions, and patient-centered approaches. The evolving landscape of dental science continually shapes the methodologies employed, underscoring the need to stay abreast of advancements to provide the highest standards of care.

The multifaceted nature of generalized attrition among older adults underscores the necessity for a holistic and detailed approach to dental care. From meticulous diagnostics to tailored interventions, ongoing maintenance, and embracing technological advancements, each step contributes to the overall success of the treatment. The journey towards optimal oral health is not merely a series of procedures; it is a commitment to the well-being and quality of life of individuals. Ensuring that they continue to smile with confidence and enjoy the pleasures of life unhindered by oral health challenges stands as the ultimate goal of comprehensive dental care.

## Conclusions

The successful management of severe tooth wear and reduced VDO in a 75-year-old patient showcases the effectiveness of the Hobo twin-stage technique. The thorough approach involving precise diagnosis, root canal therapy, and the strategic use of porcelain-fused-to-metal crowns not only restored the patient's functional capacity but also significantly improved aesthetic concerns, leading to enhanced confidence and quality of life. The Hobo twin-stage technique, chosen for its simplicity, predictability, and customization capabilities, demonstrated its efficacy in providing both functional and aesthetic outcomes. The individualized strategy addressed the challenges of generalized attrition, underscoring the importance of thorough assessment, accurate treatment planning, and consistent follow-up care. Beyond immediate success, the emphasis on regular follow-up visits and diligent oral hygiene maintenance highlights the commitment to sustained oral health.

## References

[1] Thimmappa M, Katarya V, Parekh I (2021). Philosophies of full mouth rehabilitation: a systematic review of clinical studies. J Indian Prosthodont Soc.

[2] Warreth A, Abuhijleh E, Almaghribi MA, Mahwal G, Ashawish A (2020). Tooth surface loss: a review of literature. Saudi Dent J.

[3] Mittal S, Tewari S, Goel R (2014). Esthetic and functional rehabilitation of mutilated dentition and loss of vertical dimension due to amelogenesis imperfecta. Indian J Dent.

[4] Turner KA, Missirlian DM (1984). Restoration of the extremely worn dentition. J Prosthet Dent.

[5] Dadarwal A, Sharma V, Sareen K, Vashistha DK, Madaan R (2023). Reclaiming the smile: full mouth rehabilitation of a generalized attrition patient using the hobo twin-stage technique. Cureus.

[6] Maharjan A, Joshi S, Verma A, Rimal U (2019). Rehabilitation of severely attrited teeth with Hobo twin stage technique: a case report. JNMA J Nepal Med Assoc.

[7] Davies SJ, Gray RJ, Qualtrough AJ (2002). Management of tooth surface loss. Br Dent J.

[8] Wonnacott D, Frymire E, Khan S, Green ME (2019). Physician attendance during interhospital patient transfer in Ontario: 2005-2015. Can J Rural Med.

[9] Moslehifard E, Nikzad S, Geraminpanah F, Mahboub F (2012). Full-mouth rehabilitation of a patient with severely worn dentition and uneven occlusal plane: a clinical report. J Prosthodont.

[10] Thirumurthy VR, Bindhoo YA, Jacob SJ, Kurien A, Limson KS, Vidhiyasagar P (2013). Diagnosis and management of occlusal wear: a case report. J Indian Prosthodont Soc.

[11] Christensen GJ (1986). The use of porcelain-fused-to-metal restorations in current dental practice: a survey. J Prosthet Dent.

[12] Hobo S (1991). Twin-tables technique for occlusal rehabilitation: part I--mechanism of anterior guidance. J Prosthet Dent.

[13] Hobo S (1991). Twin-tables technique for occlusal rehabilitation: part II--clinical procedures. J Prosthet Dent.

[14] Tiwari B, Ladha K, Lalit A, Naik BD (2014). Occlusal concepts in full mouth rehabilitation: an overview. J Indian Prosthodont Soc.

[15] Dadarwal A, Paliwal J, Sharma V, Jaswal S, Meena R (2022). Full mouth rehabilitation using the twin stage procedure in a patient with amelogenesis imperfecta: a case report. Cureus.

